# Supplementary material for: Patterns of Intron Gain and Loss in Fungi
Source: PLoS Biol. 2004 Nov 30;2(12):e422. doi: 10.1371/journal.pbio.0020422 (PMC532390; doi:10.1371/journal.pbio.0020422)
Supplement: Table S1 — Also available at http://genes.mit.edu/NielsenEtAl/. (4.3 MB ZIP). [file pbio.0020422.st001.zip › NielsenEtAl/html/587.html]

AN5673.1.NCU09033.1.MG08314.1.FG09884.1


```
 CLUSTAL W (1.82) Multiple Sequence Alignments - Introns Inserted


Sequence 1: MG08314.1	894 aa
Sequence 2: FG09884.1	849 aa
Sequence 3: NCU09033.1	966 aa
Sequence 4: AN5673.1	902 aa
Alignment Length: 1072 aa
Number Identitical Residues: 287 aa
Alignment Score (without introns) 15028


MG08314.1 	--------MQTDLRRGSS-------GNHQSSPVADGNRADAQNNSGDEGQPLSDSDNDLD
NCU09033.1	MAAAASGQQHHAAAGGPSPSRPPLFQFQGQQQQQQQHGPPNASVYSDDGQQSGDDDDDDD
FG09884.1 	------------MQNGAS-------KMSGDGSVE--NRPPSTEADSDEANQFHSEDEDHG
AN5673.1  	--------MPNAAIAAVP----------QQPPPSASNSAFDQDKDHSADESWSAVDSDAG
          	               . .           .      : .   .   .  :     *.* .

MG08314.1 	D-----QVDLVRNGKRKRPISVS2CEL~CKQRK0------------------~VKCDRGQ
NCU09033.1	DDNNHANDQVLPNGKRKRPLSVS2DYK0-----~------------------~VKCDRGQ
FG09884.1 	D-----HETLCRNGKRKRPVSVS2FNL~LCLQL~YKISPTLKLTLPCPDVSC0VKCDRGQ
AN5673.1  	P----AQNGSSRGLKRRRPLTVS2PTA~-----~------------------~-------
          	     ::     . **:**::**                                     

MG08314.1 	PSCGWCSRNGASCEYKERKKPGLRAGYG~RELEQRLDKLEAILRS~HQEILQTS-MDGGS
NCU09033.1	PACGWCSRNTVVCEYKERKKPGLRAGYG~RELEQRLDNLEAELKR~HADILAAHGLDLNT
FG09884.1 	PSCGWCSRNGAVCEYKERKKPGLRAGYG~RELEQRLDKLEEILRS~HAEIIHGS-LVSN-
AN5673.1  	-SLSVPSLDARLTSLGVNYASSARLN-A0IELNQAVDGVHGMVRC0AQARLIETHILQGQ
          	 : .  * :    .   .  .. * . .  **:* :* :.  ::      :    :  . 

MG08314.1 	SHLPGLHHPKAPSSVRG------------SIHSLPSD-HGTPRETQPPPFTRSDSSIRTP
NCU09033.1	S--PGSVNTRATLSINANNRNNHAPSGHRSTPSLPSNGHGTPRDTGPPPVFAHAG---PD
FG09884.1 	------TNPMFAASIRQ------------SNPSLTSE-QGTPRDTHPNLFRPSEP-VRTP
AN5673.1  	PRLSMSNDLHQQGPLSY------------SSPSEPSAAHGPS-PRNPFYFHEPSS---VP
          	.  .   .     .:              *  * .* .:*..    *  .          

MG08314.1 	QAETALFLQKPSSYPPAPSAQHHSQSMEFGLHPPTPTMRDGFQSQIGPPAGISPTNSHSH
NCU09033.1	SAERALFMHKP------------PTSADFSLGPHTPAIVHDNFQHV--------ATPQPR
FG09884.1 	QTDPALFGQKAHGSNFAPS----TQSLDFSIG-HTPKMNDGYHGQN--RSSFSSAAAPSQ
AN5673.1  	SAPR--------------------PPEPVTTSPTDMSVKNSIQNQIT-------GALPSV
          	.:                       .  .   .    : ..   :             . 

MG08314.1 	IRGTSTMSSAAYDYYNPTSTASTTSSHP-HHQSPSTMTQNQAQIASDHELPPYDLLYALV
NCU09033.1	IQPAMTPSAAVQEYYGAPRAVSHQMAGPSLTAAVLPATQGQG--GAEQELPPYGVCYALM
FG09884.1 	LS-PIQHNNATQEYYTSVQPNIHSPTAP-LSSAPGASTP-------DRDMPPYDLLYALV
AN5673.1  	TPLPHITNTTPQNDYNDNESSLKVPVNLFSNQEQSFSDP-------ELDLPPYDLLYALV
          	   .   . :  : *    .                          : ::***.: ***:

MG08314.1 	DLYFKHINTWCPILHRKMTLAALFDSPVPEETDKILLHAIVATTLRYSSDTRLTEESRRR
NCU09033.1	ELYFKHIHPWCPILNRETTKDLFFGERIPNEEEKILLHAIVATAMRFATDKMLPKESRQR
FG09884.1 	DLYFKHINTWCPILHRKTTLDSLFGPSALDETDRVLLHAIVATTMRFSTDARLTEERREH
AN5673.1  	DLYFEHLNTWCPILHRRTTLDTFFGPTPLEEADRMVLYAIVATTLRFSNDSRLTEQNRKR
          	:***:*::.*****:*. *   :*.    :* ::::*:*****::*::.*  *.:: *.:

MG08314.1 	YHDVSKKRVLLYGMENSSVKSLQALVILALDLCGAGNGPPGWNIMALITRSVVQLGLSVE
NCU09033.1	FYTASKQRVLLYGMEHSSVMSLQALVILALDLCGSSNGPPGWNIMALITRGVVQLGLAVE
FG09884.1 	YHRVSKDRVLLYGMENSSVKALQALVILALDLCGSSNGPPGWNIMALITRSVVQLGLAVE
AN5673.1  	YHDSSKQKVLLYGLENSSVKALQALVILALDLVGSSNGPPGWKLLALITRSVVQLGLAVE
          	::  **.:*****:*:*** :*********** *:.******:::*****.******:**

MG08314.1 	SNSFSVSPNFTSIYTLRAMILPEPRDFIEEESRRRLFWMVYLLDRYATISTAFDFALDDK
NCU09033.1	TNSMTVCPSYPSIYTLRAMVLPEPRDFTEEETRRRLFWMVYVLDRYATISTAFDFALDDK
FG09884.1 	VTSSTVSPHYLSIYTLRAMTLSEPRDFIEEEARRRLFWMIYLLDRYATLATAFEFALDDK
AN5673.1  	SKSTLITPLYPSIYTLRAVTLPEAESWIEDEGRRRLFWMVYLLDRYSTLATAFDFALDDK
          	 .*  : * : *******: *.*...: *:* *******:*:****:*::***:******

MG08314.1 	EIDRALPCRDDLWTKNQKVETKRFRAKGTNPQHDESTTHEYEINKPENLGAFSYYIEIVT
NCU09033.1	EIDRTLPCRNELWIKNQKVETRWFYAADDDSRLNSNSSADYQVNKPENLGAFSYYIEILG
FG09884.1 	EIDRTLPCRDDLWIKNQKVDTRWF----TTDLHSGSPGHD--IDQPENLGAFAYYIEILG
AN5673.1  	DIDRKLPCKDEFFIKNQPVETRWFQ---------SSNDRPDHLIRSENVGSFGLYVEILG
          	:*** ***::::: *** *:*: *           .      : :.**:*:*. *:**: 

MG08314.1 	ILSQIHKFLKQPVDISALSDVEQWQMRYKELDNTLTNWKFNLPGEYC--SMAKLFQ-PGS
NCU09033.1	ILSKIHKFLKQPVDISSLTDVENWQHRYRELDTMLKVWKFELPDDYS--NMAKIYEHPRV
FG09884.1 	ILSKIHKFLKQPVDISALGDVEQWQLRYKELDNMLTSWKFSLPGEFG--NMAKLFN-PG-
AN5673.1  	ILSRIHVFLKRPVDIGVLSDVEEWQATYRKLDSELTTWEFNLPTEYTYENASRAFS-GSK
          	***:** ***:****. * ***:**  *::**. *. *:*.** ::  .. :: :.    

MG08314.1 	AKTLNCGWVMLHATYHT~ATIRLHSSAAYPTTRSPIFTPSYSASQRCHSAVENIAALGEF
NCU09033.1	ARTLNCAWIMLHATYHT~AVIRLHSSAAYPTVRSPIFSPSYMASQTCRDTVNNILALGKF
FG09884.1 	SKNINCAWVMLHATYHT~AVIRLHSSAAYPTTRSPIFTPSYSASQRCHGAVENVAALGEF
AN5673.1  	HKGHHCDWVQLHSVYQT2AVIRLHSSAAYPTTRSPIFTPSYSASQRCLLAVDNIVSVTRF
          	 :  :* *: **:.*:* *.***********.*****:*** *** *  :*:*: :: .*

MG08314.1 	VLTNNLLPKLGPPFAFTLWVAARVLLVHGSTVEHKVSPQIGFFVDALKEMGRYWPVARRY
NCU09033.1	VVEYNLLSTLGPPFAFTLWVAARVLLVHGSTVERSLNPDIQFFVDTLREMGKYWPVATRY
FG09884.1 	VVNNGMLAKLGPPFAFTLWVSARLLLVHGSTVERKLSPQLPFFVESLREMGRYWPVAARY
AN5673.1  	VVNNNILDKLGPPFAFTLWVSARLLLVHGSTIAHTVSPDIVFIVDTLSQMGKYWKVAERY
          	*:  .:* .***********:**:*******: :.:.*:: *:*::* :**:** ** **

MG08314.1 	CELLQRVLDEYHDSARHGDV---GFTPSSVKILADMRRTAFDLDFLISRQPHQQTA--AA
NCU09033.1	SNLLTRVLDEYKASVSEGD----EVTPSSVRILADMRRTAFDLDLLISRQPRHGSRQHGA
FG09884.1 	SQLLQRVLDEHHDSERQGD----GITPSSVKILSDMRRTAFDLDFLISRQPKQSGG--SA
AN5673.1  	SSILQRVLDEYGEYQQSGAGDGDRSTPSSVKILADMRRCAFDLDFLISRQPRSSPT----
          	..:* *****:      *  ...  *****:**:**** *****:******:        

MG08314.1 	HGGSTGAASLAAPAGAAPTAQGFGSRYPSITPARTPQ----PNELEYLDVFDFFNYPRLP
NCU09033.1	LTGTAAGNSVMSAGVMMPVTNNSNGHFGMGTEGGTGGGGGGANDLEYLDVFDFFNMPRFP
FG09884.1 	L-----------------------SRLPSVTPARTPA----PNELEYLDVFDFFNVPRLP
AN5673.1  	------------------------ASQAAAPSAGLPSRSLAPNELEYLDVFGFFNVPRVP
          	                        .     . .     . ..*:*******.*** **.*

MG08314.1 	PGAPA-EGNANAHGNGAGNGNGEGGGGEGGGEGAAVVPSNE-------------------
NCU09033.1	LSAIAPAQNASGTETNQNHSMGQGHGMEGAGGGRGLIDDGESGSKQPPGQGMGSDVMHGS
FG09884.1 	FGG---ENISSANSTLAPPGTDMDASNDSG---TGLGASNE-------------------
AN5673.1  	AGRTPDISGLDMAEAVNNPMSIPGLTGTGSSNPLPVDNASTHTN----------------
          	 .  .     .            .    ...    :   .   .                

MG08314.1 	-------------FNITDFIMDV-NRDWLFKQ--~-------------EGTGNFVS----
NCU09033.1	AMGGGGSAQGVGQFNIMDFMVYP-ERDWLENP--~-------------RGS-SWVDATGG
FG09884.1 	-------------FNITNFMVDA-NSDWLFKQ--~-------------EGGPKYMAAT--
AN5673.1  	------------EFNITNYLIPTPETDWLFRPGV1HKSNQRIFGYIALRLNGLSCSKATS
          	            .*** ::::  .: *** . .   .:..   .  : .        : .

MG08314.1 	-----------------------------------------------------------~
NCU09033.1	RPEQGTGASGPRARTGTGSAEGAGATL--------------------------------~
FG09884.1 	-----------------------------------------------------------~
AN5673.1  	LPLIITRALVQKAKVSSGSMHPPRTAVDWQELTQEHFNFRVACTGEPLPCLPSDPAAHA1
          	 .   : :    :  .:.:   . :: . .. :..  .   :.:... .. .:..:: : 

MG08314.1 	-
NCU09033.1	-
FG09884.1 	-
AN5673.1  	V
          	
```
